# Changing epidemiology of non-occupational acute carbon monoxide poisoning and the increasing share of older adults during the clean heating transition: a 10-year emergency department study in rural Tianjin, China

**DOI:** 10.3389/fmed.2026.1919330

**Published:** 2026-08-25

**Authors:** Wei Kang, Yanqiu Du, Miao Zhao, Jisheng Yang, Jian Liang, Xin Wang, Xianjia Ning, Guohua Zhao

**Affiliations:** 1 Department of Emergency, Tianjin Jizhou People’s Hospital, Tianjin, China; 2 Institute of Clinical Epidemiology and Evidence-Based Medicine, Tianjin Jizhou People’s Hospital, Tianjin, China

**Keywords:** carbon monoxide poisoning, clean heating, emergency department, older adults, rural health

## Abstract

**Background and Objective:**

Non-occupational Acute Carbon Monoxide Poisoning (ACOP) remains a preventable emergency condition in northern rural China, where winter heating and indoor combustion have historically been important exposure sources. In rural Tianjin, the clean heating transition period was accompanied by a substantial decline in the overall incidence of non-occupational ACOP, while the residual burden shifted toward older adults and lifestyle-related exposure sources. However, whether this transition has changed the epidemiological profile of non-occupational ACOP, particularly the residual risk among older adults, remains unclear. This study aimed to examine changes in the incidence, demographic profile, exposure sources, and severity of non-occupational ACOP during the clean heating transition in rural Tianjin, China, with special attention to older adults.

**Methods:**

This single-center retrospective observational study included patients with non-occupational ACOP who presented to the Emergency Department of Tianjin Jizhou District People’s Hospital between January 2016 and December 2025. The study period was divided into a policy construction and transition period (2016–2019) and a policy implementation and consolidation period (2020–2025). Annual emergency department-attended (ED- attended) incidence rates were calculated using resident population data. Demographic characteristics, exposure sources, seasonal distribution, clinical severity, and outcomes were compared between the two periods. Negative binomial regression was used to estimate incidence rate ratios because the annual case counts were substantially overdispersed, with quasi-Poisson, robust-variance Poisson and standard Poisson models fitted as concordant sensitivity analyses, and exploratory logistic regression was performed to assess factors associated with severe poisoning, including death.

**Results:**

A total of 3,923 patients were included, with 2,241 cases during 2016–2019 and 1,682 cases during 2020–2025. The ED- attended incidence, calculated using emergency department-treated ACOP cases and resident population denominators, decreased from 0.620 to 0.356 per 1,000 population, corresponding to a 42.7% reduction during 2020–2025 (negative binomial IRR = 0.573, 95% CI: 0.432–0.759, *P* < 0.001). The demographic profile shifted toward older adults, with the proportion of patients aged ≥ 65 years increasing from 27.4 to 37.1% (*P* < 0.001). Coal stove heating remained the dominant exposure source but decreased from 94.1 to 89.8%, whereas charcoal hotpot-related poisoning increased from 4.2 to 9.1% (both *P* < 0.001). The overall severity profile showed a higher proportion of mild cases in the later period; however, severe poisoning among older adults increased from 0.8 to 2.4% (*P* = 0.040). These patterns persisted in sensitivity and heating-season restricted analyses.

**Conclusion:**

During the clean heating transition in rural Tianjin, the ED-attended incidence of non-occupational acute carbon monoxide poisoning declined substantially in both age groups, but older adults came to account for an increasing share of the remaining cases, and charcoal hotpot use accounted for a growing proportion of residual exposures. These findings suggest that clean heating consolidation should be complemented by targeted geriatric risk management, winter safety education, carbon monoxide alarm promotion, safe charcoal use, and community-based prevention strategies for older adults living in rural and semi-rural settings.

## 1 Introduction

Acute carbon monoxide poisoning (ACOP) remains a major preventable emergency condition worldwide and is particularly common in cold seasons and in regions where indoor combustion is used for heating, cooking, or other domestic activities. Because carbon monoxide is colorless, odorless, and non-irritating, exposure is often unrecognized until patients develop nonspecific symptoms such as headache, dizziness, nausea, altered consciousness, or cardiopulmonary instability. Severe ACOP may lead to coma, myocardial injury, respiratory failure, death, and delayed neuropsychiatric sequelae, imposing both acute treatment burdens and long-term functional consequences (1–4). Epidemiological evidence indicates that carbon monoxide poisoning remains an important global public health problem, with estimated cumulative incidence and mortality rates of 137 and 4.6 cases per million people, respectively (5). In northern rural China, household coal combustion for winter heating has historically been an important source of non-occupational ACOP, especially when coal stoves are used in poorly ventilated rooms during the heating season (1, 6). More broadly, winter heating-related air pollution has been associated with multiple adverse health outcomes, including respiratory diseases, cardiovascular diseases, exacerbation of chronic conditions, and cognitive decline, highlighting the wider health relevance of household energy use and heating-related environmental exposure (7). From a geriatric perspective, ACOP is not only an acute toxicological event but also a preventable environmental hazard that may disproportionately affect older adults with reduced physiological reserve, chronic disease burden, functional vulnerability, or delayed recognition of exposure.

Globally, household air pollution from incomplete combustion of solid fuels remains an important preventable environmental health risk, especially in settings where heating and cooking activities occur indoors (8). In recent years, China has implemented large-scale clean heating policies to reduce household coal combustion and improve air quality in northern regions. The national Clean Heating Plan for Northern China proposed a context-specific transition from scattered coal use to cleaner energy sources, including electricity and gas, under the principle of selecting heating technologies according to local conditions (9). Regional air pollution control policies in the Beijing–Tianjin–Hebei area further accelerated the replacement of traditional coal heating systems (10). Accumulating evidence suggests that clean heating policies have produced measurable environmental and health benefits, including improved air quality and reduced risks of pollution-related cardiopulmonary outcomes (11–13). For example, a recent quasi-experimental study in Beijing reported that the “coal-to-clean heating” policy was associated with a reduction in acute myocardial infarction incidence, supporting the broader health relevance of household energy transition (12). However, the impact of clean heating transition on non-occupational ACOP has received far less attention than its effects on ambient air pollution and chronic cardiopulmonary outcomes.

Existing epidemiological studies of non-occupational ACOP in China have described strong seasonality, household clustering, and exposure sources such as coal heating, gas leakage, charcoal combustion, and catering-related events (5, 14–17). These studies have improved understanding of the conventional epidemiological profile of ACOP, but several important questions remain unresolved. First, it is unclear whether the decline in traditional coal-related exposure after clean heating transition is accompanied by a sustained reduction in the overall incidence of non-occupational ACOP at the regional level. Second, as coal heating-related poisoning decreases, the relative contribution of other exposure sources, such as charcoal hotpot or gas-related events, may increase, suggesting a possible shift from heating-related exposure to lifestyle- or device-related exposure. Third, little is known about whether older adults remain a residual high-risk population after environmental policy transition. This issue is particularly relevant to geriatric medicine, because older adults commonly have multimorbidity, cardiopulmonary comorbidities, cognitive impairment, frailty, or limited self-care capacity, all of which may reduce tolerance to hypoxic injury and delay escape from hazardous environments (2, 18, 19). Therefore, population-level environmental interventions may not benefit all age groups equally if older adults continue to experience residual exposure risks or more severe clinical outcomes.

Tianjin Jizhou District is a rural and semi-rural area in northern China that underwent substantial clean heating transition during the past decade. The local emergency department provides an important real-world setting for evaluating changes in non-occupational ACOP during this transition. Our previous study described the epidemiological characteristics of non-occupational ACOP in Jizhou District from 2016 to 2019 (6). However, whether the epidemiological pattern changed after the implementation and consolidation of clean heating policies, and whether older adults became a more prominent residual high-risk group, remains unclear. Using a 10-year emergency department dataset from 2016 to 2025, the present study aimed to compare the incidence, demographic profile, exposure sources, and severity of non-occupational ACOP before and after clean heating transition in rural Tianjin, China. Particular attention was given to changes in the proportion of older patients and severe poisoning among older adults, with the goal of informing community-based prevention and geriatric risk management in the post-clean-heating era.

## 2 Materials and methods

### 2.1 Study design and reporting guideline

This was a single-center, retrospective observational epidemiological study based on emergency department records. The study was designed to characterize temporal changes in the incidence, demographic profile, exposure sources, and severity of non-occupational ACOP across the clean heating transition period in Jizhou District, Tianjin, China, rather than to establish a causal effect of the policy, using available official annual population data from 2016 to 2025. Particular attention was given to the residual disease burden and severe outcomes among older adults. The manuscript was prepared in accordance with the STROBE recommendations for observational studies (20).

### 2.2 Study setting and policy periods

The study was conducted at the Emergency Department of Tianjin Jizhou District People’s Hospital, a tertiary hospital serving Jizhou District, a rural and semi-rural district in northern China. The hospital is the only tertiary medical institution in the district equipped with a hyperbaric oxygen chamber and is the main regional center for the diagnosis and treatment of clinically recognized ACOP. Therefore, its emergency department records provide a meaningful real-world data source for monitoring regional non-occupational ACOP.

During the study period, Jizhou District underwent a major household energy transition in the context of the national clean heating strategy for northern China. The national clean heating plan promoted the replacement of scattered household coal combustion with cleaner energy sources, including electricity and gas, according to local conditions (9). Regional policies in the Beijing–Tianjin–Hebei area further accelerated clean heating and air pollution control measures (10). The clean heating transition was implemented gradually rather than as a single discrete event. Based on the local timeline of policy promotion, infrastructure construction, and household adaptation to new heating systems, January 2020 was selected as an analytical cut-off point to distinguish two observation periods. The period from 2016 to 2019 was defined as the policy construction and transition period, whereas 2020 to 2025 was defined as the policy implementation and consolidation period. This cut-off was used for descriptive and comparative analyses and should not be interpreted as an exact date of policy completion or as a causal intervention point.

### 2.3 Participants

All patients presenting to the Emergency Department of Tianjin Jizhou District People’s Hospital between January 1, 2016 and December 31, 2025 were screened for eligibility. Patients were included if they met all of the following criteria: (1) a diagnosis of ACOP based on clinical manifestations, exposure history, and available examination findings consistent with the Chinese expert consensus on diagnosis and treatment of ACOP (1); (2) a clear history of non-occupational carbon monoxide exposure; and (3) the poisoning event occurred within Jizhou District.

Patients were excluded if the poisoning was occupational, if the event occurred outside Jizhou District, or if the record represented a repeat visit for a previously diagnosed poisoning episode. For patients with more than one emergency visit related to the same poisoning event, only the first visit was included.

### 2.4 Data sources and data collection

Data for 2016–2019 were obtained from a previously published epidemiological investigation conducted by the same research team (6). Data for 2020–2025 were retrospectively extracted from the emergency department registration system and electronic medical records of Tianjin Jizhou District People’s Hospital. The same eligibility criteria and core variable definitions were applied across the two study periods to improve comparability.

Exposure-source information was obtained from routine emergency department records rather than from additional interviews conducted by the study team. During routine clinical care, emergency physicians and nurses recorded the suspected exposure source based on information provided by patients, family members, cohabitants, or emergency responders when available. These records usually included the suspected combustion source, exposure location, heating or cooking device involved, and relevant contextual information, such as coal stove heating, charcoal hotpot use, artificial gas leakage, gas water heater or gas appliance use, or charcoal heating or indoor barbecue. For the present study, the research team retrospectively extracted and classified exposure sources according to these documented records, together with emergency or discharge diagnoses. When the exposure source was unclear, inconsistent, or insufficiently documented, it was classified as other or unknown.

The extracted variables included year and month of presentation, age, sex, exposure source, occurrence during the heating season, living-alone status among older adults, poisoning severity, death, ICU admission, hospitalization, and treatment pathway, including only hyperbaric oxygen therapy or emergency observation. Data extraction was performed using anonymized records. When exposure source or clinical severity was not directly coded in the database, it was determined according to the documented exposure history, clinical records, and discharge or emergency diagnosis.

### 2.5 Definitions and variables

The primary exposure variable was study period, categorized as 2016–2019 and 2020–2025. Annual emergency department-attended (ED- attended) incidence was calculated as the number of non-occupational ACOP cases presenting to the Emergency Department of Tianjin Jizhou District People’s Hospital divided by the corresponding resident population and expressed per 1,000 population. Because the hospital is the only tertiary medical institution in Jizhou District equipped with a hyperbaric oxygen chamber and serves as the main regional treatment center for clinically recognized ACOP, this measure was used to approximate the regional emergency department-treated burden of non-occupational ACOP. However, it should not be interpreted as a complete population-based incidence estimate, because very mild cases not seeking medical care, cases treated outside the hospital, and pre-hospital deaths may not have been captured.

Age was analyzed both as a continuous variable and as a categorical variable. Older adults were defined as patients aged ≥ 65 years. For geriatric subgroup description, age was further classified into < 65, 65–74, 75–84, and ≥ 85 years. Sex was categorized as male or female.

Exposure source was classified according to documented exposure history, clinical records, and emergency or discharge diagnoses. Categories included coal stove heating, charcoal hotpot use, artificial gas leakage, gas water heater or gas appliance use, charcoal heating or indoor barbecue, and other or unknown sources.

The heating season was defined as November to March, corresponding to the local winter heating period and the season in which household combustion-related exposure was most likely to occur. Non-heating season was defined as April to October. Living-alone status among older adults was recorded when documented in the medical record or emergency registration information. Living arrangement was recorded opportunistically rather than ascertained systematically or prospectively, and patients whose living arrangement was not documented were classified as “not documented” rather than as not living alone. Because documentation completeness differed substantially between the two periods, this variable was reported descriptively with an explicit “not documented” category and was not used for between-period hypothesis testing or for regression modelling.

Poisoning severity was classified as mild, moderate, or severe according to the Chinese expert consensus on the diagnosis and treatment of ACOP (1). Mild poisoning was defined as ACOP with clear consciousness and symptoms such as headache, dizziness, nausea, vomiting, or limb weakness, without obvious severe complications. Moderate poisoning was defined as ACOP with transient disturbance of consciousness, confusion, or shallow coma, with recovery of consciousness after timely treatment, with or without mild neurological manifestations. Severe poisoning was defined as ACOP with deep coma or life-threatening complications, including cerebral edema, pulmonary edema, respiratory failure, myocardial injury, shock, or other severe organ dysfunction. Deaths recorded in the emergency department or hospital records were also included in the severe outcome category.

To address the potential influence of regional population aging, we performed an additional approximate age-specific incidence analysis. Official annual age-specific population data for Jizhou District were not available for individual study years; only the 2020 Seventh National Population Census (21) provided a district-level age structure, in which the population aged ≥ 65 years was 126,968 and the population aged < 65 years was 668,532, corresponding to an older-adult share of 15.96%. In the base-case analysis, age-specific person-years were obtained by applying this census age share to the official resident population of each individual study year and summing across the years of each period, so that annual changes in total population size were taken into account. Age-specific incidence rates were calculated as the number of cases in each age group divided by the corresponding person-years, and IRRs with 95% CIs were estimated for each age group; the ratio of the two age-specific IRRs was used to quantify whether the relative decline differed by age.

Because the base-case analysis holds the age structure constant, it does not capture growth of the older population between the two periods. We therefore added a scenario analysis in which the share of residents aged ≥ 65 years was allowed to increase linearly at rates of 0.2 to 1.0 percentage points per year, anchored to the observed 2020 census value; this range spans and exceeds the pace of population ageing reported nationally. For each scenario, age-specific person-years, age-specific IRRs, and the ratio of the two age-specific IRRs were recalculated, and the rate of ageing at which the two age groups would show an identical relative decline was determined. These analyses were intended to quantify the direction and magnitude of the bias introduced by the fixed age structure. Because true annual age-specific person-years were not observable, all resulting figures are approximations and are not age-standardized population-based incidence rates.

### 2.6 Outcomes

The primary outcome was the incidence rate of non-occupational ACOP during the two study periods. Secondary outcomes included demographic distribution, age structure, proportion of older adults, exposure source distribution, seasonal distribution, severity profile, death, ICU admission, hospitalization, and treatment pathway. A key geriatric outcome was severe poisoning among older adults, calculated as the proportion of severe poisoning or death among patients aged ≥ 65 years.

### 2.7 Bias control and missing data

Several measures were used to reduce potential bias. First, the study was restricted to patients with non-occupational exposure events occurring within Jizhou District to improve consistency between case ascertainment and the population denominator. Second, occupational poisoning and repeated visits for the same poisoning event were excluded to reduce misclassification and duplicate counting. Third, the study was conducted in the main regional center for ACOP diagnosis and hyperbaric oxygen therapy, which helped maintain relatively stable case capture across the study period. Fourth, the same diagnostic consensus and comparable variable definitions were applied to both periods.

Missing data were handled according to the nature of each variable. Variables required for eligibility, including exposure history, poisoning location, and diagnosis, were mandatory for inclusion. For descriptive variables that were not consistently available, analyses were conducted using available data, and the corresponding denominator was reported when applicable. No imputation was performed because most variables used in the primary analyses were categorical and derived from emergency records. Living arrangement among older adults was the variable with the largest amount of missing information, and the extent of that missingness is reported in full. Living arrangement was not documented for 565 of 613 older patients (92.2%) during 2016–2019 and for 480 of 624 (76.9%) during 2020–2025, and no older patient during 2016–2019 was affirmatively documented as not living alone. Because documentation completeness differed markedly between periods and appeared to be driven by the presence rather than the absence of the characteristic, undocumented patients were reported as a separate category rather than being combined with patients documented as not living alone, and no between-period comparison of the prevalence of living alone was performed.

### 2.8 Statistical analysis

Continuous variables were summarized as mean ± standard deviation when approximately normally distributed and compared using independent-samples t tests. Categorical variables were summarized as numbers and percentages and compared using chi-square tests. Fisher’s exact test was used when expected cell counts were small. For ordered or multi-category variables, including severity classification and age groups, contingency table chi-square tests were used. For proportions based on small numbers of events, such as severe poisoning among older adults, exact 95% confidence intervals were calculated using the Clopper–Pearson method to quantify statistical uncertainty.

Annual incidence rates were calculated per 1,000 population. IRRs and 95% CIs comparing the two study periods were estimated from annual case counts, with the logarithm of the annual resident population included as the offset. Overdispersion was assessed using the Pearson dispersion statistic, which indicated substantial overdispersion in the annual counts (Pearson χ^2^/df = 22.5, *P* < 0.001). The principal period comparison was therefore estimated using negative binomial regression rather than standard Poisson regression, and quasi-Poisson regression and Poisson regression with robust (sandwich) standard errors were fitted as concordant alternatives (Supplementary Table 1). The official annual resident population data were used for 2016–2024, and the 2024 resident population was used as the denominator for 2025 because official 2025 population data were not yet available. The percentage change in incidence between adjacent years was calculated to describe annual fluctuation.

Exploratory logistic regression was performed to identify factors associated with severe poisoning including death. Variables included in the model were study period, age group, sex, heating season, and charcoal hotpot use. Detailed exposure-source categories were not simultaneously entered into the multivariable model because several categories had no severe events, which could lead to complete separation and unstable estimates. Crude ORs and adjusted ORs with 95% CIs were reported. The multivariable logistic regression model was exploratory and included variables that were available with acceptable completeness and consistency across the study period. Important clinical and social factors, including underlying cardiovascular disease, cerebrovascular disease, chronic lung disease, cognitive impairment, living arrangement, COHb level, mode of presentation, exposure duration, and treatment delay, were not included because they were not systematically recorded in the retrospective emergency department records. Therefore, the model was intended to describe associations rather than to establish independent causal predictors of severe poisoning.

Two additional analyses were conducted to assess the robustness of the findings. First, to reduce the potential influence of COVID-19-related changes in healthcare-seeking behavior, referral patterns, social activity, and reporting practices, a sensitivity analysis was performed by excluding the pandemic period from 2020 to 2022 and comparing 2016–2019 with the post-pandemic later period of 2023–2025. Second, a heating-season restricted analysis was performed because most non-occupational ACOP cases occurred during the local winter heating period.

To evaluate temporal patterns beyond the two-period comparison, segmented negative binomial regression was performed. Non-occupational ACOP in this district is confined almost entirely to the 5-month winter heating season: only 79 of 2,241 cases (3.5%) during 2016–2019 and 109 of 1,682 (6.5%) during 2020–2025 occurred outside it, corresponding to fewer than two cases per non-heating month on average. A monthly series therefore consists of a small number of extreme winter peaks separated by near-zero months, and monthly indicator variables or Fourier harmonics fitted to such a series would be estimated largely from months containing no cases while representing what is essentially a 5-month on/off pattern as a smooth periodic cycle. The analysis was therefore conducted at the two temporal resolutions these data support.

First, annual case counts were modelled over the ten study years with the logarithm of the annual resident population as the offset. Aggregation to the calendar year removes the within-year seasonal cycle by construction, so that no additional seasonal term is required. The model included calendar time as a continuous variable, an interruption indicator coded 0 before 2020 and 1 from 2020 onward, and post-interruption time increasing by one unit per year thereafter. Second, the same segmented specification was fitted to a year-by-season panel of twenty observations, in which the annual counts were divided into heating-season (November to March) and non-heating-season (April to October) strata, a heating-season indicator was entered explicitly, and the offset was the corresponding person-time, calculated as five twelfths and seven twelfths of the annual resident population respectively. An extended version of this model additionally included a heating season-by-period interaction term to test whether the decline differed between seasons. Residual serial correlation was assessed in both models using the Durbin–Watson statistic and the Ljung–Box portmanteau test at lags 1 to 3, applied to the Pearson residuals ordered by calendar time within each season stratum. Regression coefficients were exponentiated and reported as IRRs with 95% CIs (Supplementary Table 2). These analyses were conducted as exploratory sensitivity analyses describing temporal patterns and were not intended to establish a causal policy effect.

All statistical tests were two-sided, and *P* < 0.05 was considered statistically significant. Statistical analyses were performed using SPSS version 25.0.

### 2.9 Ethics statement

This study was based on retrospective review of routinely collected and de-identified emergency department records. The study protocol was reviewed and approved by the Ethics Committee of Tianjin Jizhou People’s Hospital (IRB2026-32). Because the study used anonymized retrospective data and did not involve direct patient contact, the requirement for written informed consent was waived by the ethics committee.

## 3 Results

### 3.1 Overall temporal trend in ED-attended non-occupational ACOP from 2016 to 2025

A total of 3,923 ED-attended patients with non-occupational ACOP were included during the 10-year study period. Of these, 2,241 cases occurred during the policy construction and transition period from 2016 to 2019, and 1,682 cases occurred during the policy implementation and consolidation period from 2020 to 2025.

Using annual resident population data from 2016 to 2025, the annual ED-attended incidence rate ranged from 0.501 to 0.760 per 1,000 population during 2016–2019 and from 0.239 to 0.494 per 1,000 population during 2020–2025. The highest annual incidence was observed in 2018, with 685 cases and an incidence rate of 0.760 per 1,000 population. A marked decline was observed between 2019 and 2020, around the analytical cut-off point, when the ED-attended incidence decreased from 0.709 to 0.317 per 1,000 population, corresponding to a 55.3% reduction. Thereafter, the incidence fluctuated at a lower level and further decreased to 0.239 per 1,000 population in 2025.

**FIGURE 1:**
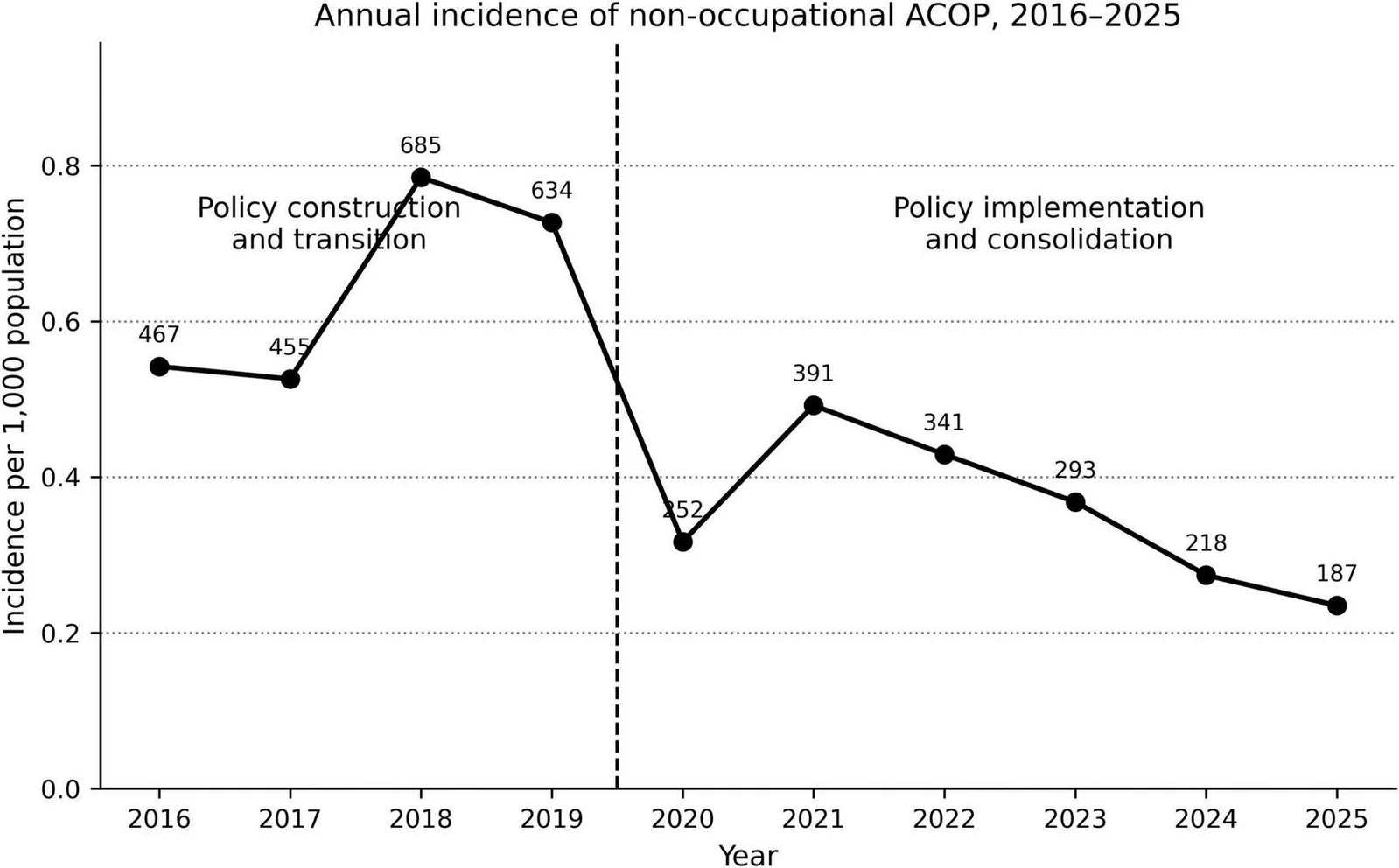
Annual ED-attended incidence of non-occupational ACOP from 2016 to 2025. This figure showed that the overall ED-attended incidence rate decreased from 0.62 per 1,000 population during 2016–2019 to 0.36 per 1,000 population during 2020–2025. The dashed vertical line indicates January 2020, which was used as an analytical cut-off point to distinguish the policy construction and transition period from the policy implementation and consolidation period. Because the clean heating transition was implemented gradually, this cut-off should not be interpreted as an exact date of policy completion or as evidence of a causal intervention effect.

The ED-attended incidence rate decreased from 0.62 per 1,000 population during 2016–2019 to 0.36 per 1,000 population during 2020–2025. The annual case counts were substantially overdispersed (Pearson χ^2^/df = 180.2/8 = 22.5, *P* < 0.001). Negative binomial regression showed that the ED-attended incidence rate during 2020–2025 was 42.7% lower than that during 2016–2019 (IRR = 0.573, 95% CI: 0.432–0.759, *P* < 0.001). Quasi-Poisson regression (IRR = 0.574, 95% CI: 0.425–0.774, *P* < 0.001) and Poisson regression with robust standard errors (IRR = 0.574, 95% CI: 0.439–0.751, *P* < 0.001) yielded essentially identical point estimates. The corresponding standard Poisson interval (IRR = 0.574, 95% CI: 0.539–0.611) was considerably narrower and is not reported as the principal estimate because it does not account for overdispersion (Table 1; Figure 1 and Supplementary Table 1).

**TABLE 1:** Annual incidence of non-occupational ACOP in Jizhou District, Tianjin, 2016–2025.

Study period	Year	No. of cases	Resident population	Incidence rate per 1,000 population	Annual percentage change, %
Policy construction and transition period	2016	467	911,500	0.512	Reference
	2017	455	908,400	0.501	−2.2
	2018	685	901,900	0.760	51.6
	2019	634	894,500	0.709	−6.7
Policy implementation and consolidation period	2020	252	795,500	0.317	−55.3
	2021	391	790,800	0.494	56. 1
	2022	341	788,700	0.432	−12. 6
	2023	293	792,100	0.370	−14.4
	2024	218	782,100	0.279	−24.6
	2025	187	782,100	0.239	−14.2
2016–2019	Overall	2,241	3,616,300	0.620	Reference
2020–2025	Overall	1,682	4,731,300	0.356	−42.6
Statistical comparison				IRR = 0.574, negative binomial IRR = 0.573 (95% CI: 0.432–0.759)	*P* < 0.001

### 3.2 Demographic shift and increasing residual burden among older adults

The demographic profile of patients changed significantly between the two periods. The proportion of male patients increased from 35.3% during 2016–2019 to 43.7% during 2020–2025, whereas the proportion of female patients decreased from 64.7 to 56.3% (χ^2^ = 28.224, *P* < 0.001). The mean age of patients increased from 51.3 ± 18.5 years to 55.3 ± 18.7 years (t = 6.721, *P* < 0.001).

**FIGURE 2:**
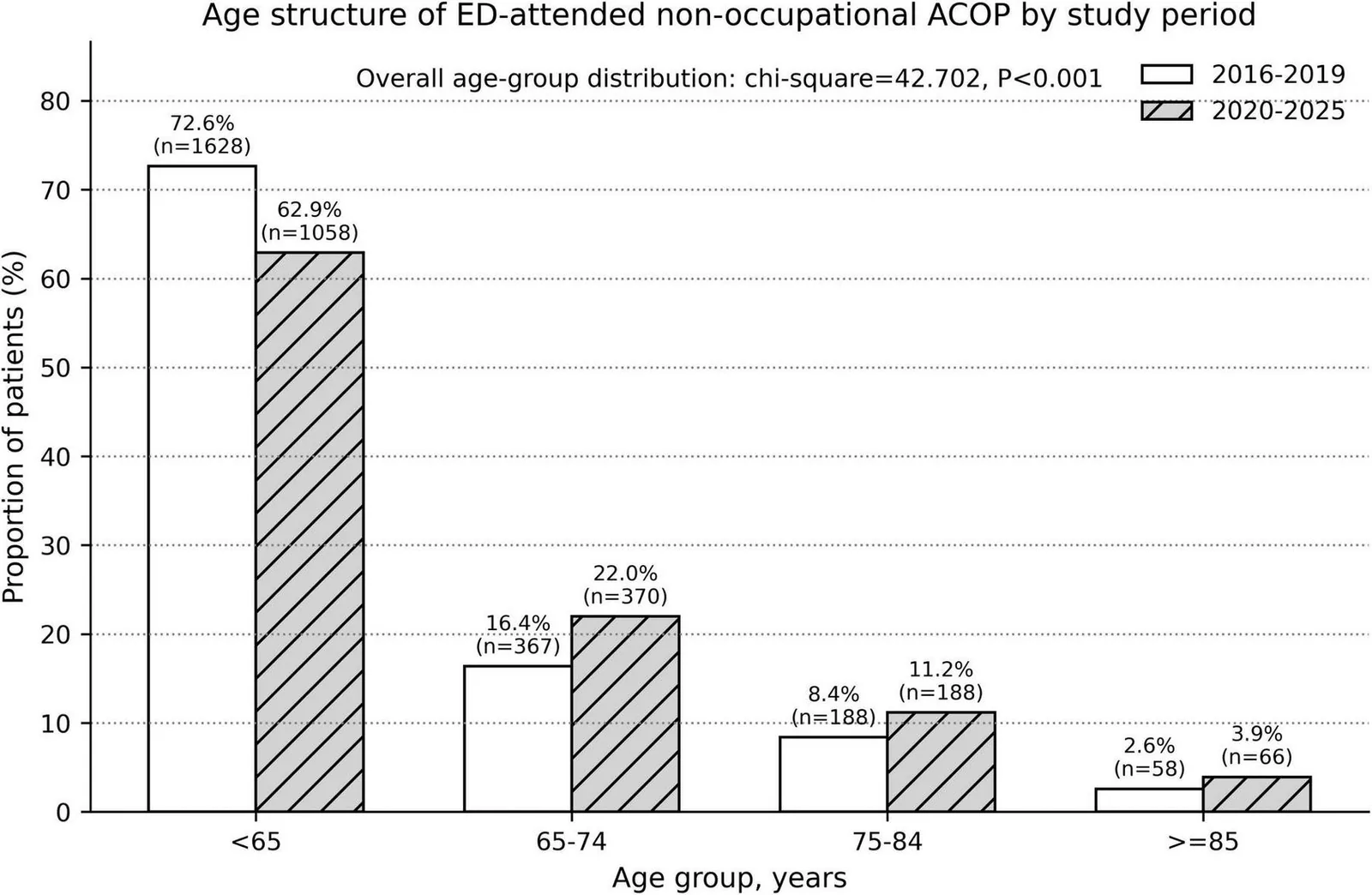
Age structure of patients with non-occupational acute carbon monoxide poisoning by study period. The overall age-group distribution differed significantly between 2016–2019 and 2020–2025 (χ^2^ = 42.702, *P* < 0.001). Percentages represent the proportion of patients in each age group within each study period.

A clear shift toward an older age structure was observed. The proportion of patients aged ≥ 65 years increased from 613/2,241 (27.4%) during 2016–2019 to 624/1,682 (37.1%) during 2020–2025. The overall age-group distribution differed significantly between the two periods (χ^2^ = 42.702, *P* < 0.001). This shift was observed across all older age strata: the proportions of patients aged 65–74, 75–84, and ≥ 85 years increased from 16.4 to 22.0%, from 8.4 to 11.2%, and from 2.6 to 3.9%, respectively (Figure 2 and Table 2).

**TABLE 2:** Demographic characteristics of patients with non-occupational ACOP by study period.

Variables	2016–2019 (*n* = 2,241)	2020–2025 (*n* = 1,682)	Test statistic	*P*-value
Sex, *n* (%)			χ^2^ = 28.224	< 0.001
Male	792 (35.3)	735 (43.7)		
Female	1,449 (64.7)	947 (56.3)		
Age, years	51.3 ± 18.5	55.3 ± 18.7	*t* = 6.721	< 0.001
Age group, *n* (%)			χ^2^ = 42.702	< 0.001
< 65 years	1,628 (72.6)	1,058 (62.9)		
65–74 years	367 (16.4)	370 (22.0)		
75–84 years	188 (8.4)	188 (11.2)		
≥ 85 years	58 (2.6)	66 (3.9)		
Heating season cases, *n* (%)	2,162 (96.5)	1,573 (93.5)	χ^2^ = 18.391	< 0.001
Non-heating season cases, *n* (%)	79 (3.5)	109 (6.5)		
Living arrangement among older adults, *n* (%)				
Living alone	48 (7.8)	98 (15.7)		
Not living alone	0 (0.0)	46 (7.4)		
Not documented	565 (92.2)	480 (76.9)		
Living arrangement documented, *n* (%)	48 (7.8)	144 (23.1)	χ^2^ = 54.820	< 0.001
Living alone among patients with documented status, *n/N* (%)	48/48 (100.0)	98/144 (68.1)	Fisher exact test	< 0.001

Denominators for the living-arrangement rows are all patients aged ≥ 65 years in each period (613 during 2016–2019 and 624 during 2020–2025). Living arrangement was recorded only when documented in the emergency record and was not ascertained systematically; patients without documentation are shown as a separate “not documented” category rather than being counted as not living alone. Documentation was markedly more complete during 2020–2025 (144/624, 23.1%) than during 2016–2019 (48/613, 7.8%), and no older patient during 2016–2019 was affirmatively documented as not living alone, indicating that living arrangement was recorded almost exclusively when the patient was living alone. Among patients with a documented living arrangement the proportion living alone was higher in the earlier period, the opposite direction to that obtained when all older patients are used as the denominator. The distribution shown therefore reflects differential documentation and cannot be interpreted as an estimate of the prevalence of living alone in either period; accordingly, no between-period comparison of that prevalence is reported and living arrangement was not entered into any regression model (Supplementary Table 4).

To further account for the potential influence of regional population aging, we estimated age-specific ED-attended incidence by applying the 2020 census age structure to the resident population of each individual study year. Among patients aged < 65 years, the estimated ED-attended incidence decreased from 0.536 per 1,000 person-years during 2016–2019 to 0.266 per 1,000 person-years during 2020–2025 (IRR = 0.497, 95% CI: 0.460–0.537), a 50.3% reduction. Among patients aged ≥ 65 years, it decreased from 1.062 to 0.826 per 1,000 person-years (IRR = 0.778, 95% CI: 0.696–0.870), a 22.2% reduction. The ratio of these two age-specific IRRs was 1.566 (95% CI: 1.368–1.794, *P* < 0.001), indicating that the estimated ED-attended incidence fell in both age groups but less steeply among older adults (Table 3).

**TABLE 3:** Estimated age-specific ED-attended incidence of non-occupational ACOP by study period.

Age group	Study period	Person-years	Cases	Rate per 1,000 person-years	IRR (95% CI)	Relative reduction, %
< 65 years	2016–2019	3,039,110	1,628	0.536	Reference	–
	2020–2025	3,976,148	1,058	0.266	0.497 (0.460–0.537)	50.3
≥ 65 years	2016–2019	577,190	613	1.062	Reference	–
	2020–2025	755,152	624	0.826	0.778 (0.696–0.870)	22.2
Ratio of age-specific IRRs ( ≥ 65 vs. < 65 years)					1.566 (1.368–1.794)	*P* < 0.001

Person-years were obtained by applying the age structure of the 2020 Seventh National Population Census for Jizhou District (15.96% aged ≥ 65 years) to the official resident population of each individual study year and summing across the years of each period. Because official annual age-specific denominators were not available, these rates are approximations and are not age-standardized population-based incidence rates. The ratio of the two age-specific IRRs compares the relative decline among patients aged ≥ 65 years with that among patients aged < 65 years; a value > 1 indicates a smaller relative decline among older adults.

Because this calculation holds the age structure constant, it does not capture growth of the older population, and the direction of the resulting bias works against the observed difference: if the share of residents aged ≥ 65 years increased over time, the true decline among older adults would be larger and that among younger adults smaller than estimated. In the scenario analysis, the ratio of age-specific IRRs fell from 1.566 under a constant age structure to 1.252 if the older-adult share rose by 0.6 percentage points per year and to 1.161 if it rose by 0.8 percentage points per year, and would reach 1.0 only if the share rose by approximately 1.19 percentage points per year, a pace substantially faster than that reported nationally (Supplementary Table 3). The smaller relative decline among older adults therefore does not appear to be explained by population ageing alone. Nevertheless, because annual age-specific denominators were not observable, these figures remain approximations, and the finding that is most directly supported by the data is the increase in the share of older adults among the remaining cases rather than an increase in their absolute risk.

Most cases occurred during the heating season in both periods. However, the proportion of heating-season cases decreased from 96.5% during 2016–2019 to 93.5% during 2020–2025, whereas the proportion of non-heating-season cases increased from 3.5 to 6.5% (χ^2^ = 18.391, *P* < 0.001).

Living arrangement among older adults was documented for only a minority of patients, and documentation completeness differed markedly between the two periods. Living arrangement was documented for 48 of 613 older patients (7.8%) during 2016–2019 and for 144 of 624 (23.1%) during 2020–2025 (χ^2^ = 54.820, *P* < 0.001). No older patient during 2016–2019 was affirmatively documented as not living alone, whereas 46 were so documented during 2020–2025, indicating that in the earlier period living arrangement was recorded almost exclusively when the patient was living alone. Among older patients with a documented living arrangement, 48 of 48 (100.0%) during 2016–2019 and 98 of 144 (68.1%) during 2020–2025 were living alone (Fisher’s exact test, *P* < 0.001), a difference in the opposite direction to that obtained when all older patients are used as the denominator. Because the direction of the comparison depends entirely on how undocumented patients are handled, these data cannot support any inference about a change in the prevalence of living alone between the two periods; living arrangement is therefore reported descriptively with an explicit “not documented” category and was not entered into any regression model (Table 2 and Supplementary Table 4).

### 3.3 Changes in exposure sources across the study periods

The distribution of exposure sources changed significantly between the two periods (overall comparison: χ^2^ = 42.032, *P* < 0.001). Coal stove heating remained the dominant source of non-occupational ACOP, but its proportion decreased from 94.1% during 2016–2019 to 89.8% during 2020–2025, corresponding to a relative decrease of 4.5% (*P* < 0.001). In contrast, the proportion of cases attributed to charcoal hotpot use increased from 4.2 to 9.1%, corresponding to a relative increase of 116.9% (*P* < 0.001). Other exposure sources, including artificial gas leakage, gas water heater or gas appliance use, and charcoal heating or indoor barbecue, accounted for only a small proportion of cases. These findings indicate that, over the study period, the residual exposure profile shifted from an almost exclusive dominance of traditional coal stove heating toward a greater relative contribution of lifestyle-related charcoal combustion, particularly charcoal hotpot use (Table 4).

**TABLE 4:** Changes in exposure sources of non-occupational ACOP by study period: composition and source-specific ED-attended incidence.

Exposure source	2016–2019, *n* (%)	2020–2025, *n* (%)	Relative change in proportion, %	Rate per 1,000 person-years, 2016–2019	Rate per 1,000 person-years, 2020–2025	IRR for rate (95% CI)	*P*-value for rate
Coal stove heating	2,109 (94.1)	1,511 (89.8)	−4.5	0.583	0.319	0.548 (0.513–0.585)	< 0.001
Charcoal hotpot use	94 (4.2)	153 (9.1)	+116.9	0.026	0.032	1.244 (0.962–1.608)	0.108
Artificial gas leakage	28 (1.2)	11 (0.7)	−47.7	0.0077	0.0023	0.300 (0.149–0.603)	< 0.001
Gas water heater / gas appliance	4 (0.2)	3 (0.2)	−0.1	0.0011	0.0006	0.573 (0.128–2.561)	0.476
Charcoal heating / indoor barbecue	6 (0.3)	4 (0.2)	−11.2	0.0017	0.0008	0.510 (0.144–1.806)	0.347
All sources	2,241 (100)	1,682 (100)	–	0.620	0.356	0.573 (0.432–0.759)	< 0.001

Percentages are column percentages within each study period. Relative change in proportion is the change in the column percentage between the two periods, expressed relative to the earlier period; the overall distribution of exposure sources differed significantly between periods (χ^2^ = 42.032, *P* < 0.001). Rates are the number of cases attributed to each source divided by the total person-years of the corresponding period (3,616,300 person-years in 2016–2019 and 4,731,300 person-years in 2020–2025). A change in the proportion of cases attributable to a source does not imply a corresponding change in the source-specific rate: the proportion attributable to charcoal hotpot use increased substantially, whereas the charcoal hotpot-specific rate did not increase significantly. For all sources combined, the IRR shown is the negative binomial estimate accounting for overdispersion (Supplementary Table 1).

Because these figures describe the composition of cases rather than population-based risk, exposure-source–specific ED-attended incidence rates were also calculated. The coal stove-related rate decreased from 0.583 to 0.319 per 1,000 person-years (IRR = 0.548, 95% CI: 0.513–0.585, *P* < 0.001), a 45.2% reduction. The rate of poisoning attributed to artificial gas leakage also decreased, from 0.0077 to 0.0023 per 1,000 person-years (IRR = 0.300, 95% CI: 0.149–0.603, *P* < 0.001). In contrast, the charcoal hotpot-related rate changed little, from 0.026 to 0.032 per 1,000 person-years (IRR = 1.244, 95% CI: 0.962–1.608, *P* = 0.108), and the remaining sources were too infrequent for stable estimation. The near-doubling of the charcoal hotpot proportion therefore reflects predominantly the marked decline in coal stove-related cases rather than a demonstrable increase in the population rate of charcoal hotpot-related poisoning (Table 4).

### 3.4 Severity profile and severe outcomes among older adults

The overall severity distribution differed significantly between the two periods (χ^2^ = 8.822, *P* = 0.012). Mild cases accounted for a higher proportion during 2020–2025 than during 2016–2019, whereas moderate cases decreased. Severe poisoning, including deaths, remained uncommon but increased from 0.9 to 1.5%. The overall number of deaths was low and did not differ significantly between the two periods.

In terms of treatment pathway and severe outcomes, ICU admission increased from 0.5 to 1.2% (*P* = 0.024), while the proportion of patients managed with only hyperbaric oxygen therapy or emergency observation increased from 78.8 to 81.3% (*P* = 0.047).

Among older adults, severe poisoning increased from 5/613 (0.8%; exact 95% CI: 0.3%–1.9%) during 2016–2019 to 15/624 (2.4%; exact 95% CI: 1.4%–3.9%) during 2020–2025 (Fisher’s exact test, *P* = 0.040). Although this difference was statistically significant, the absolute number of events was small and the confidence intervals overlapped, indicating limited statistical stability. Therefore, this finding should be interpreted cautiously (Table 5).

**TABLE 5:** Severity profile and severe poisoning among older adults by study period.

Variables	2016–2019 (*n* = 2,241)	2020–2025 (*n* = 1,682)	Test statistic	*P*-value
Overall severity, *n* (%)			χ^2^ = 8.822	0.012
Mild	1,765 (78.8)	1,368 (81.3)		
Moderate	456 (20.3)	289 (17.2)		
Severe, including deaths	20 (0.9)	25 (1.5)		
Deaths, *n* (%)	8 (0.4)	5 (0.3)	Fisher exact test	0.788
ICU admission, *n* (%)	12 (0.5)	20 (1.2)	χ^2^ = 5.073	0.024
General Hospitalization, *n* (%)	468 (20.9)	309 (18.4)	χ^2^ = 3.819	0.051
Only Hyperbaric oxygen therapy/Emergency observation, n (%)	1,765 (78.8)	1,368 (81.3)	χ^2^ = 3.953	0.047
Severe poisoning among older adults	5/613 (0.8)	15/624 (2.4)	Fisher exact test	0.040
Deaths among older adults	2/613 (0.3)	2/624 (0.3)	Fisher exact test	1.000

ICU admission, general hospitalization, and hyperbaric oxygen therapy/emergency observation are not mutually exclusive categories; a patient may belong to more than one category (e.g., admitted to the ICU and subsequently transferred to a general ward). Severe poisoning includes deaths.

### 3.5 Factors associated with severe poisoning, including death

Exploratory logistic regression was performed to identify factors associated with severe poisoning, including death. In crude analyses, the odds of severe poisoning were higher during 2020–2025 than during 2016–2019, but the association did not reach statistical significance (crude OR = 1.675, 95% CI: 0.927–3.027, *P* = 0.087). Patients aged ≥ 65 years also had higher crude odds of severe poisoning than those aged < 65 years, with borderline statistical significance (crude OR = 1.749, 95% CI: 0.968–3.162, *P* = 0.064). Sex, heating season, and charcoal hotpot use were not significantly associated with severe poisoning in crude analyses.

After adjustment for study period, age group, sex, heating season, and charcoal hotpot use, none of the included variables remained statistically significant. The adjusted OR was 1.593 (95% CI: 0.875–2.900, *P* = 0.128) for 2020–2025 versus 2016–2019 and 1.612 (95% CI: 0.878–2.960, *P* = 0.124) for patients aged ≥ 65 years versus those aged < 65 years (Table 6). These findings suggest a possible but statistically non-significant tendency toward higher severe poisoning risk in the later period and among older adults. Because the number of severe events was small, several detailed exposure-source categories had zero severe events, and several clinically important variables were unavailable or inconsistently recorded, the multivariable model should be interpreted as exploratory and may be affected by residual confounding.

**TABLE 6:** Factors associated with severe poisoning, including death, among patients with non-occupational ACOP.

Variables	OR (95% CI)	*P*-value
Unadjusted:		
2020–2025 vs. 2016–2019	1.675 (0.927–3.027)	0.087
≥ 65 years vs. < 65 years	1.749 (0.968–3.162)	0.064
Male vs. female	1.259 (0.697–2.274)	0.446
Heating season vs. non-heating season	1.083 (0.260–4.505)	0.913
Charcoal hotpot use vs. other sources	0.690 (0.166–2.864)	0.609
Adjusted:		
2020–2025 vs. 2016–2019	1.593 (0.875–2.900)	0.128
≥ 65 years vs. < 65 years	1.612 (0.878–2.960)	0.124
Male vs. female	1.166 (0.642–2.116)	0.614
Heating season vs. non-heating season	0.342 (0.017–6.818)	0.482
Charcoal hotpot use vs. other sources	0.312 (0.016–6.197)	0.445

### 3.6 Segmented regression analysis of temporal trends

The annual case counts were substantially overdispersed relative to a Poisson distribution (Pearson χ^2^/df = 180.2/8 = 22.5, *P* < 0.001), and negative binomial models were therefore used throughout. In the annual segmented regression, the ED-attended incidence was rising before the interruption point (IRR = 1.150 per year, 95% CI: 1.003–1.319, *P* = 0.046). A downward level change was observed at 2020 (IRR = 0.659, 95% CI: 0.443–0.981, *P* = 0.040), and the post-interruption slope was significantly lower than the pre-interruption slope (IRR = 0.787, 95% CI: 0.671–0.922, *P* = 0.003), indicating that the incidence both fell abruptly and continued to decline more steeply thereafter.

Seasonality was pronounced. In the year-by-season panel model the heating-season rate was more than twenty times the non-heating-season rate (IRR = 27.328, 95% CI: 19.162–38.976, *P* < 0.001). After explicit adjustment for season, the level change at 2020 remained significant (IRR = 0.456, 95% CI: 0.229–0.907, *P* = 0.025), whereas the post-interruption slope change was attenuated and no longer significant (IRR = 0.819, 95% CI: 0.622–1.079, *P* = 0.156). When a heating season-by-period interaction was added it was significant (IRR = 0.492, 95% CI: 0.261–0.930, *P* = 0.029): the ED-attended rate decreased from 1.435 to 0.798 per 1,000 heating-season person-years (IRR = 0.556) but changed little outside the heating season, from 0.037 to 0.039 per 1,000 non-heating-season person-years (IRR = 1.055). The decline was therefore concentrated in the heating season.

Residual serial correlation was not detected in either model. In the annual model the Durbin–Watson statistic was 2.13 and the Ljung–Box test was non-significant at lags 1 to 3 (*Q* = 0.12, *P* = 0.729; *Q* = 2.79, *P* = 0.247; *Q* = 2.82, *P* = 0.420). In the year-by-season model the Durbin–Watson statistic was 1.25 and the Ljung–Box test was likewise non-significant (*Q* = 1.52, *P* = 0.218; *Q* = 3.02, *P* = 0.221; *Q* = 4.61, *P* = 0.203). The estimated dispersion parameters were 0.022 and 0.126 respectively. The segmented regression results were therefore directionally consistent with the two-period comparison and were not attributable to unmodelled seasonality or serial correlation (Supplementary Table 2).

### 3.7 Sensitivity analysis excluding the COVID-19 pandemic period

To reduce the potential influence of COVID-19-related changes in healthcare-seeking behavior and social activity, a sensitivity analysis was conducted after excluding the pandemic period from 2020 to 2022. This analysis compared the pre-pandemic/pre-interruption period of 2016–2019 with the post-pandemic later period of 2023–2025. During 2023–2025, 698 cases were recorded, and the incidence rate was 0.296 per 1,000 population. Compared with 2016–2019, the incidence rate during 2023–2025 was substantially lower (IRR = 0.478, 95% CI: 0.439–0.520, *P* < 0.001).

The age shift persisted in this sensitivity analysis. The mean age increased from 51.3 ± 18.5 years during 2016–2019 to 56.0 ± 18.3 years during 2023–2025 (*t* = 5.934, *P* < 0.001), and the proportion of older adults increased from 613/2,241 (27.4%) to 264/698 (37.8%) (χ^2^ = 27.860, *P* < 0.001). Coal stove heating-related poisoning decreased from 94.1 to 86.8% (χ^2^ = 40.176, *P* < 0.001), whereas charcoal hotpot-related poisoning increased from 4.2 to 11.9% (χ^2^ = 55.705, *P* < 0.001). Severe poisoning among older adults increased from 5/613 (0.8%) to 7/264 (2.7%), showing a persistent upward tendency, although the difference was of borderline statistical significance (Fisher’s exact test, *P* = 0.051) (Supplementary Table 5).

### 3.8 Heating-season restricted analysis

Because most cases occurred during the heating season, a heating-season restricted analysis was performed. During the heating season, 2,162 cases occurred in 2016–2019 and 1,573 cases occurred in 2020–2025. The incidence rate decreased from 1.435 to 0.798 per 1,000 heating-season person-years, corresponding to a 44.4% lower incidence during 2020–2025 (IRR = 0.556, 95% CI: 0.521–0.593, *P* < 0.001).

Among heating-season cases, the proportion of older adults increased from 608/2,162 (28.1%) to 621/1,573 (39.5%) (χ^2^ = 53.191, *P* < 0.001). Coal stove heating remained the dominant exposure source, but its proportion decreased from 2,109/2,162 (97.5%) to 1,511/1,573 (96.1%) (χ^2^ = 6.775, *P* = 0.009). Charcoal hotpot-related poisoning increased from 18 cases (0.8%) to 49 cases (3.1%) (χ^2^ = 26.927, *P* < 0.001). Severe poisoning also increased from 18 cases (0.8%) to 25 cases (1.6%) (χ^2^ = 4.582, *P* = 0.032). Among older adults during the heating season, severe poisoning increased from 5/608 (0.8%) to 15/621 (2.4%) (Fisher’s exact test, *P* = 0.040) (Supplementary Table 6).

## 4 Discussion

### 4.1 Summary of principal findings

In this 10-year emergency department-based study, we examined temporal changes in the epidemiology of non-occupational ACOP across the clean heating transition period in rural Tianjin, with particular attention to residual risk among older adults. The main findings were as follows. First, the overall ED-attended incidence of non-occupational ACOP declined substantially during 2020–2025 compared with 2016–2019. Because this was a single-region, single-center, before-and-after study based on emergency department records without an external control region, this decline should be interpreted as a temporal change in ED-attended ACOP rather than direct evidence of a causal effect of the clean heating policy or true population-based incidence. Second, the demographic profile shifted toward an older age structure, with patients aged ≥ 65 years accounting for an increasing proportion of residual cases. Third, the exposure profile changed: coal stove heating remained the dominant source but decreased in proportion, whereas charcoal hotpot-related poisoning increased notably. Fourth, although the overall severity profile showed a higher proportion of mild cases, severe poisoning among older adults increased significantly, suggesting that older adults may remain a vulnerable subgroup for adverse outcomes despite the decline in overall emergency department presentations.

### 4.2 Comparison with previous studies

The seasonal and household-related nature of non-occupational ACOP has been well established, whereas evidence on epidemiological changes across the clean heating transition period remains limited. Previous studies from northern and eastern China consistently showed that non-occupational ACOP was highly concentrated in winter and was mainly associated with household coal combustion, gas leakage, charcoal use, and poor indoor ventilation (6, 14–17). From a broader public health perspective, incomplete combustion of household fuels remains an important preventable source of poisoning and indoor environmental risk (8, 22, 23). In parallel, clean heating policies in northern China have been associated with improved air quality and reduced pollution-related health risks, including cardiopulmonary outcomes (8, 10–13). However, most policy evaluations have focused on ambient air pollution or chronic cardiopulmonary diseases rather than acute poisoning events. In this context, our finding that the overall ED-attended incidence of non-occupational ACOP declined markedly during 2020–2025 provides real-world evidence that the clean heating transition period coincided with a substantial reduction in emergency department-treated ACOP. Because this was a single-region, single-center, before-and-after study without an external control region, this decline should be interpreted as a temporal association rather than direct evidence of a causal policy effect.

Our findings should also be interpreted in the broader context of regional and global changes in household energy use and carbon monoxide poisoning prevention. Previous studies from Beijing, Shanghai, and other regions of China have shown that non-occupational carbon monoxide poisoning remains strongly seasonal and is associated with household coal combustion, gas leakage, charcoal use, and catering-related exposure sources. International evidence also indicates that carbon monoxide poisoning remains a preventable public health problem linked to indoor combustion, fuel-burning appliances, poor ventilation, and delayed recognition of exposure. Therefore, the decline observed in Jizhou District is broadly consistent with the expected public health benefits of reducing scattered household coal combustion, but it cannot be attributed solely to the clean heating transition without an external control region.

The shift in exposure sources across the study period remains an important finding. Earlier epidemiological studies reported coal heating as the dominant source of winter ACOP, while urban studies from Beijing and Shanghai also identified gas leakage, charcoal combustion, catering-related exposure, and restaurant events as important non-occupational sources (14–17). Clinical and public health guidance also emphasizes that carbon monoxide can be generated by multiple household and recreational sources, including coal, gas appliances, charcoal grills, and poorly ventilated combustion devices (22–24). Consistent with these reports, coal stove heating remained the predominant source in our study, but its proportion decreased, whereas charcoal hotpot-related poisoning increased substantially during the later period. This pattern suggests that the residual exposure profile may be shifting from traditional heating-related exposure toward lifestyle-related charcoal combustion. Possible explanations include changes in household heating practices, indoor dining behaviors, enclosed restaurant or home dining environments, and reduced ventilation during winter. However, these interpretations should be considered exploratory because the present study could not directly measure household behavior, ventilation conditions, or policy implementation intensity.

The increasing proportion of older adults among residual ACOP cases should be interpreted in the context of regional population aging. Most prior reports focused on overall case counts, seasonality, geographic distribution, and exposure sources, with less attention to age-related vulnerability (6, 14–17). In our additional age-specific incidence analysis, the estimated incidence decreased in both age groups, suggesting that the absolute risk of emergency department-treated ACOP did not increase among older adults. However, the decline was smaller among patients aged ≥ 65 years than among those aged < 65 years. Two caveats apply to this comparison. First, annual age-specific denominators were unavailable, so the age-specific rates rest on a census-based age structure applied to observed annual populations. Second, the direction of the resulting bias works against the observed difference, and the scenario analysis indicated that the difference would be eliminated only under a pace of population ageing far exceeding national figures. Even so, the observation that is most directly supported by these data is the rising share of older adults among the remaining cases; a rising absolute risk among older adults is not demonstrated. From a geriatric perspective, this pattern remains clinically important because older adults often have high chronic disease burden, multimorbidity, frailty, reduced physiological reserve, and impaired functional capacity (18, 19, 25–27). These characteristics may increase susceptibility to hypoxic injury and reduce the ability to recognize or escape from hazardous environments. Potential contributing factors may include long-standing heating habits, difficulty adapting to new heating devices, limited safety awareness, financial concerns about operating costs, and social vulnerability such as living alone.

The increase in severe poisoning among older adults is a clinically relevant but exploratory finding. Prior clinical studies have shown that ACOP can cause myocardial injury, coma, delayed neuropsychiatric sequelae, and long-term neurological impairment (2–4, 24, 28–30). Clinical practice recommendations emphasize that ACOP severity is influenced not only by exposure intensity but also by patient susceptibility, comorbid conditions, delayed recognition, and timely removal from the exposure source (23, 24). In our study, severe poisoning among older adults increased from 0.8 to 2.4% during the later period. However, this finding was based on a small number of events, and the exact confidence intervals overlapped. This pattern may reflect residual vulnerability among older adults with poorer hypoxic tolerance, underlying cardiopulmonary or cerebrovascular disease, delayed discovery, or limited ability to leave the exposure environment. Therefore, it should be interpreted as an exploratory signal rather than definitive evidence of increased severe risk. Nevertheless, the finding suggests that older adults may remain vulnerable to severe outcomes even when overall ED-attended ACOP incidence declines. Larger multicenter studies are needed to validate this observation and to clarify whether comorbidity, frailty, living alone, delayed rescue, or exposure intensity contributes to severe outcomes among older adults.

The persistence of a strong heating-season pattern is consistent with previous studies, but our findings suggest that seasonal prevention strategies should be updated. Previous reports from Jizhou, Beijing, Shanghai, and national risk assessments all indicated that non-occupational ACOP occurs predominantly in winter, largely because heating activities, closed windows, and poor ventilation increase indoor carbon monoxide accumulation (6, 14–17). International public health guidance similarly highlights winter heating, fuel-burning appliances, charcoal grills, and inadequate ventilation as major preventable sources of household carbon monoxide exposure (22, 23, 31). Our heating-season restricted analysis showed that incidence remained lower during 2020–2025 than during 2016–2019, while the proportion of older adults increased and charcoal hotpot-related poisoning became more frequent. These findings suggest that winter prevention should not focus only on coal stove replacement. It should also address safe charcoal hotpot use, adequate ventilation in homes and restaurants, carbon monoxide alarms, and targeted winter safety checks for older adults living alone or with limited self-care capacity.

The annual fluctuations observed during the study period should be interpreted cautiously. The higher case counts in 2018–2019 may reflect a combination of year-to-year variation in winter temperature, heating duration, household stove use, ventilation practices, population activity patterns, and case capture during the transition period. Because annual meteorological data, household ventilation conditions, and individual-level heating behavior were not systematically available, we could not directly determine the drivers of this increase. Similarly, the marked single-year decline in 2020 should not be attributed solely to the clean heating transition. This decrease may have reflected the combined influence of clean heating consolidation and COVID-19-related changes in healthcare-seeking behavior, referral patterns, mobility, social activities, household exposure patterns, and reporting practices. For this reason, we performed a sensitivity analysis excluding 2020–2022, and the lower ED-attended incidence and shifts in age structure and exposure sources persisted when 2016–2019 was compared with 2023–2025. These findings support the robustness of the overall temporal pattern but do not establish a causal policy effect.

The exploratory interrupted time-series analysis showed a lower level estimate after the interruption point and a non-significant downward post-interruption trend, which was directionally consistent with the two-period comparison. However, the ITS estimates were not statistically significant, likely reflecting strong seasonality, substantial monthly variation, and limited statistical power. Therefore, the observed decline should be interpreted as a temporal pattern rather than causal evidence of policy effectiveness.

### 4.3 Strengths and limitations

The main strength of this study is its 10-year emergency department-based dataset from the main regional center for ACOP diagnosis and hyperbaric oxygen therapy in Jizhou District. This allowed us to evaluate temporal changes before and after the clean heating transition using a consistent clinical data source. In addition, the study focused not only on overall incidence but also on age structure, exposure sources, and older adult-specific severe outcomes.

Several limitations should be noted. First, this was a single-center retrospective study based on emergency department records rather than a complete population-based surveillance system. Although Tianjin Jizhou District People’s Hospital is the main regional treatment center for clinically recognized ACOP and the only tertiary hospital in the district equipped with a hyperbaric oxygen chamber, some cases may not have been captured. Mild cases may not have sought medical care, may have been treated in primary care settings, or may have gone to other hospitals. Conversely, some severe cases may have died before reaching the emergency department. In addition, changes in emergency care accessibility, referral patterns, public awareness, and hyperbaric oxygen treatment capacity over time may have affected case capture. Therefore, the ED-attended incidence reported in this study should be interpreted as a hospital-based estimate of emergency department-treated ACOP and may represent a lower-bound estimate of the true regional burden. Although we estimated age-specific ED-attended incidence using the 2020 census age structure as a fixed reference population, annual age-specific population denominators were not publicly available for each study year. Therefore, these estimates should be interpreted as approximate age-specific ED-attended incidence rates rather than fully age-standardized population-based incidence rates. Second, the before-and-after design without an external control region limits causal inference. Although the decline in ED-attended non-occupational ACOP occurred during the clean heating transition period, it cannot be directly attributed to the clean heating policy. Other time-varying factors may also have influenced emergency department presentations, including changes in healthcare-seeking behavior during the COVID-19 pandemic, population mobility, household activity patterns, climate variation, healthcare accessibility, public awareness, and reporting practices. In addition, we did not have a comparable control region with similar demographic and rural characteristics but without clean heating transition. Therefore, the observed changes should be interpreted as temporal associations rather than causal effects. Future studies using external control regions, interrupted time-series analysis, or difference-in-differences designs are needed to better evaluate the causal contribution of clean heating policies to changes in non-occupational ACOP incidence. Third, official resident population data for 2025 were not available; therefore, the 2024 resident population was used as the denominator for estimating the 2025 incidence rate. Fourth, the retrospective nature of the study limited the availability and consistency of several clinically, socially, and environmentally important variables. Underlying cardiovascular disease, cerebrovascular disease, chronic lung disease, cognitive impairment, functional status, frailty, medication use, living arrangement, COHb level, mode of presentation, exposure duration, treatment delay, detailed household heating practice, ventilation condition, carbon monoxide alarm use, and individual participation in the clean heating transition process were not systematically recorded. These factors may influence both exposure risk and clinical severity. Therefore, the exploratory regression model could not fully adjust for residual confounding, and the mechanisms underlying residual exposure and severe poisoning among older adults could not be directly evaluated. In addition, the segmented regression analysis was constrained by the temporal resolution that these data support. Because non-occupational ACOP is confined almost entirely to the 5-month heating season, seasonality could be adjusted for only as a binary heating-season contrast rather than through monthly indicator variables or harmonic terms, and the models were fitted to ten annual observations and to a twenty-observation year-by-season panel. The small number of time points limits the precision of the level and slope estimates. The absence of an external control region and the coincidence of the interruption point with the onset of the COVID-19 pandemic further limit causal interpretation, so the segmented regression should be regarded as a description of temporal pattern rather than as evidence of a causal policy effect. Finally, the number of severe events was small, and the multivariable regression analysis should therefore be interpreted as exploratory. In particular, the observed increase in severe poisoning among older adults was based on only 5 and 15 events in the two periods, respectively; therefore, this finding should be regarded as exploratory and requires validation in larger multicenter datasets. Living arrangement among older adults deserves particular mention. It was undocumented for 92.2% of older patients during 2016–2019 and for 76.9% during 2020–2025, and no older patient during the earlier period was affirmatively documented as not living alone, indicating that the characteristic was recorded when present rather than assessed in every patient. Because documentation completeness improved over time, the observed distribution of living arrangement cannot be interpreted as a valid estimate of its prevalence in either period, and the between-period comparison reported in the previous version has been withdrawn. Social vulnerability such as living alone therefore remains a plausible but untested hypothesis in this dataset rather than a demonstrated finding, and prospective recording of living arrangement will be required before it can be evaluated.

## 5 Conclusion

In this single-center emergency department-based study from rural Tianjin, the emergency department-attended incidence of non-occupational acute carbon monoxide poisoning decreased substantially from 2016–2019 to 2020–2025. However, older adults accounted for an increasing proportion of residual cases, and the decline in estimated age-specific emergency department-attended incidence was smaller among patients aged ≥ 65 years than among those aged < 65 years. Although coal stove heating remained the dominant exposure source, charcoal hotpot-related poisoning contributed a greater relative share in the later period. These findings support continued winter prevention strategies that combine clean heating consolidation with safe charcoal use, ventilation improvement, carbon monoxide alarm promotion, and targeted protection of older adults.

## Data Availability

The raw data supporting the conclusions of this article will be made available by the authors, without undue reservation.
