# Supplementary material for: Changing epidemiology of non-occupational acute carbon monoxide poisoning and the increasing share of older adults during the clean heating transition: a 10-year emergency department study in rural Tianjin, China
Source: Front Med (Lausanne). 2026 Aug 25;13:1919330. doi: 10.3389/fmed.2026.1919330 (PMC13550967; doi:10.3389/fmed.2026.1919330)
Supplement: Supplementary file 1 [file Data_Sheet_1.docx]

**Supplementary Table 1. Period comparison of ED-attended non-occupational ACOP incidence under alternative count models**

| **Model** | **IRR (2020–2025 vs 2016–2019)** | **95% CI** | **P value** | **Overdispersion accounted for** |
| --- | --- | --- | --- | --- |
| Poisson, pooled counts per period (previous version) | 0.574 | 0.539–0.611 | <0.001 | No |
| Poisson, annual counts with population offset | 0.574 | 0.539–0.611 | <0.001 | No |
| Quasi-Poisson, annual counts | 0.574 | 0.425–0.774 | <0.001 | Yes |
| Negative binomial, annual counts (α=0.047) | 0.573 | 0.432–0.759 | <0.001 | Yes |
| Poisson with robust (sandwich) standard errors | 0.574 | 0.439–0.751 | <0.001 | Yes |

All models used the logarithm of the resident population as the offset. The Pearson dispersion statistic for the annual counts was 180.2/8 = 22.5 (P<0.001), indicating substantial overdispersion. Point estimates were essentially identical across models; the confidence intervals from the models that account for overdispersion are appropriately wider. The negative binomial estimate is reported as the principal estimate in the main text.

**Supplementary Table 2. Segmented negative binomial regression of ED-attended non-occupational ACOP incidence, 2016–2025**

| **Model / parameter** | **IRR** | **95% CI** | **P value** |
| --- | --- | --- | --- |
| Model A: annual counts (10 annual observations) |  |  |  |
| Pre-interruption annual trend | 1.150 | 1.003–1.319 | 0.046 |
| Level change at 2020 | 0.659 | 0.443–0.981 | 0.040 |
| Post-interruption slope change | 0.787 | 0.671–0.922 | 0.003 |
| Dispersion parameter α | 0.022 | — | — |
| Durbin–Watson statistic | 2.13 | — | — |
| Ljung–Box test, lags 1 / 2 / 3 | — | Q=0.12 / 2.79 / 2.82 | 0.729 / 0.247 / 0.420 |
| Model B: year-by-season panel (20 observations) |  |  |  |
| Pre-interruption annual trend | 1.268 | 0.995–1.617 | 0.055 |
| Level change at 2020 | 0.456 | 0.229–0.907 | 0.025 |
| Post-interruption slope change | 0.819 | 0.622–1.079 | 0.156 |
| Heating season vs non-heating season | 27.328 | 19.162–38.976 | <0.001 |
| Dispersion parameter α | 0.126 | — | — |
| Durbin–Watson statistic | 1.25 | — | — |
| Ljung–Box test, lags 1 / 2 / 3 | — | Q=1.52 / 3.02 / 4.61 | 0.218 / 0.221 / 0.203 |
| Model C: Model B plus heating season-by-period interaction |  |  |  |
| Pre-interruption annual trend | 1.298 | 1.039–1.622 | 0.022 |
| Level change at 2020 | 0.733 | 0.353–1.522 | 0.404 |
| Post-interruption slope change | 0.780 | 0.607–1.003 | 0.053 |
| Heating season vs non-heating season | 41.274 | 25.258–67.446 | <0.001 |
| Heating season-by-period interaction | 0.492 | 0.261–0.930 | 0.029 |
| Dispersion parameter α | 0.094 | — | — |

All models are negative binomial segmented regressions. Model A uses the ten annual case counts with the logarithm of the annual resident population as the offset; aggregation to the calendar year removes the within-year seasonal cycle by construction. Models B and C use a year-by-season panel in which each year contributes a heating-season (November to March) and a non-heating-season (April to October) observation, with person-time calculated as five twelfths and seven twelfths of the annual resident population respectively. Each model includes calendar time, an interruption indicator coded 0 before 2020 and 1 from 2020 onwards, and post-interruption time. Monthly indicator variables and Fourier harmonic terms were not fitted because fewer than two cases per month occur outside the heating season, so such terms would be estimated largely from months containing no cases. Residual serial correlation was assessed using the Durbin–Watson statistic and the Ljung–Box portmanteau test applied to the Pearson residuals, ordered by calendar time within each season stratum; neither model showed significant residual autocorrelation. The dispersion parameter α quantifies overdispersion relative to a Poisson distribution.

**Supplementary Table 3. Scenario analysis of the influence of population ageing on estimated age-specific ED-attended incidence**

| **Annual increase in ≥65 share, percentage points** | **≥65 share, 2016, %** | **≥65 share, 2025, %** | **Reduction, <65 years, %** | **Reduction, ≥65 years, %** | **Ratio of age-specific IRRs** |
| --- | --- | --- | --- | --- | --- |
| 0 (base case: constant age structure) | 15.96 | 15.96 | 50.3 | 22.2 | 1.566 |
| 0.2 | 15.16 | 16.96 | 49.7 | 26.9 | 1.454 |
| 0.3 | 14.76 | 17.46 | 49.4 | 29.2 | 1.401 |
| 0.4 | 14.36 | 17.96 | 49.1 | 31.4 | 1.349 |
| 0.5 | 13.96 | 18.46 | 48.8 | 33.5 | 1.300 |
| 0.6 | 13.56 | 18.96 | 48.5 | 35.6 | 1.252 |
| 0.8 | 12.76 | 19.96 | 47.9 | 39.5 | 1.161 |
| 1.0 | 11.96 | 20.96 | 47.3 | 43.3 | 1.076 |

The share of residents aged ≥65 years was assumed to increase linearly at the stated rate, anchored to the 2020 Seventh National Population Census value of 15.96% for Jizhou District, and was applied to the official resident population of each individual study year to obtain age-specific person-years. A ratio of age-specific IRRs greater than 1 indicates a smaller relative decline among older adults. The two age groups would show an identical relative decline only if the share aged ≥65 years increased by approximately 1.19 percentage points per year, a pace substantially faster than that reported nationally. These figures quantify the direction and magnitude of the bias introduced by holding the age structure constant; they are not age-standardised population-based incidence rates.

**Supplementary Table 4. Documentation of living arrangement among patients aged ≥65 years, by year and study period**

| **Year** | **Patients aged ≥65 years, n** | **Living alone, n** | **Not living alone, n** | **Not documented, n** | **Living arrangement documented, %** |
| --- | --- | --- | --- | --- | --- |
| 2016 | 116 | 13 | 0 | 103 | 11.2 |
| 2017 | 126 | 7 | 0 | 119 | 5.6 |
| 2018 | 188 | 7 | 0 | 181 | 3.7 |
| 2019 | 183 | 21 | 0 | 162 | 11.5 |
| 2020 | 84 | 19 | 9 | 56 | 33.3 |
| 2021 | 148 | 20 | 13 | 115 | 22.3 |
| 2022 | 128 | 17 | 7 | 104 | 18.8 |
| 2023 | 117 | 22 | 8 | 87 | 25.6 |
| 2024 | 87 | 14 | 5 | 68 | 21.8 |
| 2025 | 60 | 6 | 4 | 50 | 16.7 |
| 2016–2019, total | 613 | 48 | 0 | 565 | 7.8 |
| 2020–2025, total | 624 | 98 | 46 | 480 | 23.1 |

Living arrangement was recorded only when documented in the emergency record. No patient aged ≥65 years was affirmatively documented as not living alone in any year between 2016 and 2019, whereas such documentation appears in every year from 2020 onwards, indicating a change in recording practice that coincides with the boundary between the two study periods. Documentation completeness was 48/613 (7.8%) during 2016–2019 and 144/624 (23.1%) during 2020–2025 (χ²=54.820, P<0.001). Among patients with a documented living arrangement, the proportion living alone was 48/48 (100.0%) during 2016–2019 and 98/144 (68.1%) during 2020–2025 (Fisher's exact test, P<0.001). Because the direction of the between-period difference reverses according to whether undocumented patients are excluded or treated as not living alone, no inference about a change in the prevalence of living alone can be drawn from these data.

**Supplementary Table 5. Sensitivity analysis excluding the COVID-19 pandemic period: comparison between 2016–2019 and 2023–2025**

| **Variables** | **2016–2019** | **2023–2025** | **Test statistic / IRR** | **P value** |
| --- | --- | --- | --- | --- |
| No. of cases | 2,241 | 698 | - | - |
| Incidence rate per 1,000 population | 0.620 | 0.296 | IRR=0.478 | <0.001 |
| Mean age, years | 51.3±18.5 | 56.0±18.3 | t=5.934 | <0.001 |
| Older adults, n (%) | 613 (27.4) | 264 (37.8) | χ²=27.860 | <0.001 |
| Coal stove heating, n (%) | 2,109 (94.1) | 606 (86.8) | χ²=40.176 | <0.001 |
| Charcoal hotpot use, n (%) | 94 (4.2) | 83 (11.9) | χ²=55.705 | <0.001 |
| Severe poisoning among older adults | 5/613 (0.8) | 7/264 (2.7) | Fisher exact test | 0.051 |

**Supplementary Table 6. Heating-season restricted analysis**

| **Variables** | **Heating season 2016–2019** | **Heating season 2020–2025** | **Test statistic / IRR** | **P value** |
| --- | --- | --- | --- | --- |
| No. of cases | 2162 | 1573 |  |  |
| Incidence rate per 1,000 population | 1.435 | 0.798 | IRR=0.556 | <0.001 |
| Older adults, n (%) | 608 (28.1) | 621 (39.5) | χ²=53.191 | <0.001 |
| Coal stove heating, n (%) | 2,109 (97.5) | 1,511 (96.1) | χ²=6.775 | 0.009 |
| Charcoal hotpot use, n (%) | 18 (0.8) | 49 (3.1) | χ²=26.927 | <0.001 |
| Severe poisoning, n (%) | 18 (0.8) | 25 (1.6) | χ²=4.582 | 0.032 |
| Severe poisoning among older adults | 5/608 (0.8) | 15/621 (2.4) | Fisher exact test | 0.040 |
